# A non-radioactive modified immunoprecipitation assay for diagnostic detection of anti-MDA5 antibodies in idiopathic inflammatory myopathies

**DOI:** 10.3389/fimmu.2026.1896104

**Published:** 2026-07-15

**Authors:** Tian-De Wang, Kai-Fa Teo, Joung-Liang Lan, Jye-Lin Hsu

**Affiliations:** 1 Graduate Institute of Biomedical Sciences, China Medical University, Taichung, Taiwan; 2 College of Medicine, China Medical University, Taichung, Taiwan; 3 Rheumatology and Immunology Center, China Medical University Hospital, Taichung, Taiwan; 4 Rheumatic Diseases Research Center, China Medical University Hospital, Taichung, Taiwan; 5 Drug Development Center, China Medical University, Taichung, Taiwan

**Keywords:** anti-MDA5 antibody, dermatomyositis, diagnostic specificity, idiopathic inflammatory myopathies, immunoprecipitation assay, interstitial lung disease, line blot assay

## Abstract

**Introduction:**

Anti-melanoma differentiation-associated gene 5 (MDA5) autoantibodies are clinically important in idiopathic inflammatory myopathies (IIM), but currently used screening assays may yield clinically misleading false-positive results. We aimed to compare commonly used anti-MDA5 detection methods and to evaluate a modified, non-radioactive immunoprecipitation assay as a practical confirmatory method for anti-MDA5 testing.

**Methods:**

We analysed 93 individuals, including 55 patients with IIM, 18 non-IIM cases with line-blot anti-MDA5 positivity, and 20 healthy controls. Anti-MDA5 antibodies were assessed using line blot assay, immunocytochemistry, radioimmunoassay and a modified immunoprecipitation assay using activated THP-1 cell lysates. Assay performance and agreement were evaluated using radioimmunoassay as the reference method.

**Results:**

Radioimmunoassay identified substantial discrepancies in anti-MDA5 classification by routine screening assays. Among 55 patients with IIM, 5 of 37 cases initially classified as MDA5-positive by line blot (13.5%) were false positives, and all 18 non-IIM line-blot anti-MDA5-positive cases were likewise unconfirmed by radioimmunoassay. Across all 93 individuals, line blot and immunocytochemistry showed false-positive rates of 38.33% and 21.67%, respectively. In contrast, the modified immunoprecipitation assay showed complete concordance with radioimmunoassay (kappa = 1.0), with no false-positive or false-negative results.

**Conclusion:**

False-positive anti-MDA5 results are common with line blot and immunocytochemistry, particularly in clinically ambiguous cases. The modified immunoprecipitation assay showed complete concordance with radioimmunoassay in this cohort and may provide a clinically useful non-radioactive confirmatory approach for anti-MDA5 antibody detection.

## Introduction

Idiopathic inflammatory myopathies (IIM), including polymyositis (PM), dermatomyositis (DM) and clinically amyopathic dermatomyositis (CADM), are a heterogeneous group of autoimmune diseases characterised by muscle involvement and extra-muscular manifestations (1, 2). CADM is distinguished by the presence of characteristic cutaneous features of DM with minimal or absent clinical muscle involvement (3). Interstitial lung disease (ILD) is a major complication of PM/DM/CADM and contributes substantially to morbidity and mortality (4–9).

Autoantibodies play important roles in the pathogenesis and clinical evaluation of several autoimmune diseases, including systemic lupus erythematosus, rheumatoid arthritis and Sjögren’s syndrome (10–13). In IIM, autoantibody profiling has become central to disease classification, phenotype definition and clinical management (14, 15). Melanoma differentiation-associated gene 5 (MDA5) is a cytosolic sensor of viral RNA involved in innate immune activation, although the pathogenic role of anti-MDA5 antibodies remains incompletely understood (16). Anti-MDA5 antibodies are strongly associated with ILD, especially rapidly progressive ILD (RP-ILD), in patients with DM and CADM. Given that RP-ILD is associated with high mortality and poor clinical outcomes, anti-MDA5 antibodies are important biomarkers for clinical risk stratification and prognostic assessment (7, 17–21).

Despite their clinical relevance, accurate laboratory detection of anti-MDA5 antibodies remains challenging. Commercial line blot assays, including the EUROLINE Myopathies Ag kit, are widely used for screening, but false-positive anti-MDA5 results have been reported, particularly in patients with suspected IIM and in those with other autoimmune diseases (22–27). Because anti-MDA5 status may influence disease classification, interpretation of ILD risk, and subsequent clinical decision-making, assay specificity is critical. A reliable confirmatory approach is therefore needed when screening results are discordant with the overall clinical phenotype.

In this study, we performed a comparative evaluation of commonly used anti-MDA5 detection methods and developed a modified, non-radioactive immunoprecipitation assay. We assessed its agreement with radioimmunoassay and its potential role as a practical confirmatory method for anti-MDA5 testing in patients with IIM, clinically suspected cases and healthy controls.

## Materials and methods

### Patients and line blot assay

This retrospective assay-comparison study included selected plasma samples from 55 patients with IIM, 18 non-IIM patients with line-blot anti-MDA5 positivity, and 20 healthy controls collected at China Medical University Hospital, Taiwan, between December 2015 and September 2022. The cohort was not designed as a consecutive, unselected IIM cohort, but as an assay-comparison cohort enriched for cases relevant to anti-MDA5 antibody detection. This study was approved by the Ethics Committee of China Medical University Hospital in Taiwan (No. CMUH104-REC3-093). All participants were of Taiwanese origin. Healthy controls were recruited from hospital staff members and students and had no known diagnosis of IIM or other autoimmune disease at the time of sample collection.

Final clinical diagnoses were assigned by experienced rheumatologists based on clinical manifestations, laboratory findings, myositis-specific and myositis-associated autoantibody profiles, pulmonary evaluation and available imaging studies. Patients with IIM, including DM and PM, were classified according to the 2017 EULAR/ACR classification criteria for adult and juvenile idiopathic inflammatory myopathies and their major subgroups (28). CADM was defined as characteristic cutaneous manifestations of DM with absent or minimal clinical muscle involvement for at least 6 months (29). ILD was diagnosed based on compatible clinical manifestations and radiological findings, including chest imaging or high-resolution computed tomography when available. RP-ILD was defined as acute or subacute worsening of respiratory symptoms with radiological and/or physiological deterioration within 3 months of respiratory symptom onset.

Non-IIM patients with line-blot anti-MDA5 positivity were defined as patients who showed anti-MDA5 positivity by commercial line-blot assay but did not fulfill the final clinical diagnosis of IIM, DM, PM or CADM after rheumatologic evaluation. Some of these patients also had clinical features overlapping with anti-MDA5-associated disease, including pulmonary, cutaneous, articular or vascular/vasculitic manifestations (30).

Anti-MDA5 antibodies were initially assessed using the EUROLINE Autoimmune Inflammatory Myopathies 16 Ag (IgG) kit (DL 1530-1601–4 G) according to the manufacturer’s instructions.

### Cell culture and induction of endogenous MDA5 expression

THP-1 and HeLa cells were maintained in RPMI 1640 and Dulbecco’s Modified Eagle Medium, respectively, supplemented with 10% fetal bovine serum and 1% penicillin-streptomycin at 37 °C in a humidified incubator containing 5% CO_2_. Cells were routinely tested for mycoplasma contamination using the MycoAlert kit (Lonza). For preparation of endogenous MDA5-containing lysates, THP-1 cells were seeded at approximately 6-7 × 10^6^ cells per 10-cm dish and differentiated with phorbol 12-myristate 13-acetate (PMA; InvivoGen) at a final concentration of 0.1 µg/mL for 24 h. After PMA stimulation, the PMA-containing medium was removed and replaced with fresh medium. Cells were then stimulated with LPS-EB from Escherichia coli O111:B4 (InvivoGen) at a final concentration of 1 µg/mL for 16 h to induce endogenous MDA5 expression.

### Preparation of THP-1 cell lysates

After LPS stimulation, THP-1 cells were washed 1–2 times with ice-cold PBS and lysed in 1% IGEPAL CA-630 lysis buffer containing 1% IGEPAL CA-630 (Sigma), 150 mM NaCl (Sigma), 20 mM Tris-HCl pH 8.0 (Sigma), 5 mM EDTA (Thermo Fisher Scientific), and freshly added cOmplete EDTA-free protease inhibitor cocktail (Roche). Cells were incubated in lysis buffer for approximately 5 min, and lysates were collected into microcentrifuge tubes. Lysates were clarified by centrifugation at 14,000 rpm for 15 min at 4 °C, and the supernatants were transferred to new tubes without disturbing the pellet. A part of clarified lysate was reserved as input control, and the remaining pooled lysate was used for pre-clearing.

### Preparation of protein A/G beads and lysate pre-clearing

Protein A beads (GE Healthcare Life Sciences/Cytiva) and Protein G beads (GeneTex) were mixed at equal volumes to prepare a Protein A/G bead mixture. Before use, the bead mixture was washed three times with ice-cold PBS. After the final wash, the beads were resuspended in ice-cold PBS to the original calculated slurry volume and used immediately for lysate pre-clearing and immunoprecipitation capture.

Clarified pooled THP-1 lysate was pre-cleared by incubation with washed Protein A/G bead mixture for the entire pooled lysate preparation. Pre-clearing was performed by rotation at 4 °C for 20 min. Beads were then pelleted by centrifugation at 6,000 rpm for 1 min at 4 °C, and the pre-cleared lysate supernatant was transferred to a new tube without aspirating the beads. For each immunoprecipitation reaction, 100 µL of pre-cleared THP-1 lysate, corresponding to lysate prepared from approximately 1 × 10^6^ cells, was used.

### Modified immunoprecipitation followed by immunoblotting

Plasma samples were mixed thoroughly and clarified by centrifugation at 14,000 rpm for 5 min at 4 °C to remove particulate material. For each reaction, 25 µL of clarified plasma was incubated with pre-cleared THP-1 lysate corresponding to approximately 1 × 10^6^ cells. Before immunoprecipitation, the lysate was diluted in IGEPAL-free dilution buffer containing 150 mM NaCl, 20 mM Tris-HCl pH 8.0, 5 mM EDTA, and freshly added cOmplete EDTA-free protease inhibitor cocktail, reducing the final IGEPAL CA-630 concentration to approximately 0.1%.

The plasma-lysate mixture was incubated by rotation at 4 °C for 150 min to allow circulating antibodies to bind their corresponding antigens. Washed Protein A/G bead mixture was then added, followed by an additional 30 min of rotation at 4 °C to capture antibody-antigen complexes. After immunoprecipitation, the beads were washed four times with 0.1% IGEPAL CA-630 wash buffer and eluted in sample dye by heating at approximately 95 °C for 10 min.

Immunoprecipitated proteins were separated by Bis-Tris SDS-PAGE and transferred onto polyvinylidene difluoride membranes. Recombinant human MDA5 protein (OriGene Technologies; cat. no. TP315661) was used as a positive protein control. Immunoprecipitated MDA5 was detected using a rabbit monoclonal anti-MDA5 antibody (clone D74E4; Cell Signaling Technology; cat. no. 5321), followed by an anti-rabbit IgG-Fc fragment cross-adsorbed secondary antibody (Bethyl Laboratories/Fortis Life Sciences; cat. no. A120-211P). Chemiluminescent signals were developed using Immobilon Western Chemiluminescent HRP substrate and captured with a ChemiDoc MP imaging system (Bio-Rad).

### Radioimmunoassay

For radioimmunoassay, the same immunoprecipitation, Protein A/G bead-capture, washing, and elution procedures described above were used, with modifications in antigen preparation and signal detection. To generate radiolabelled endogenous MDA5 antigen, THP-1 cells were differentiated with PMA and stimulated with LPS-EB from *Escherichia coli* O111:B4 in the presence of [^35^S]methionine protein labelling mix (EasyTag EXPRESS; Revvity/PerkinElmer). The resulting radiolabelled THP-1 cell lysates were used as the antigen source for immunoprecipitation with patient plasma.

Briefly, THP-1 cells were seeded in 10-cm dishes at approximately 6–7 × 10^6^ cells per dish and differentiated with PMA at a final concentration of 0.1 µg/mL for 24 h. Cells were then stimulated with LPS-EB at a final concentration of 1 µg/mL and metabolically labelled with 200 µCi per dish of [^35^S]methionine protein labelling mix for 16 h. After radiolabelling, all subsequent steps involving radioactive materials were performed according to institutional radiation safety procedures. Cells were washed with ice-cold PBS, lysed in 1% IGEPAL CA-630 lysis buffer, clarified by centrifugation, pre-cleared with Protein A/G beads, and used immediately for immunoprecipitation.

For each radiolabelled immunoprecipitation reaction, pre-cleared radiolabelled THP-1 lysate corresponding to approximately 1 × 10^6^ cells was incubated with 25 µL of clarified plasma at 4 °C for 150 min, followed by Protein A/G bead capture for 30 min. Beads were washed four times with 0.1% IGEPAL CA-630 wash buffer and eluted in sample dye by heating at approximately 95 °C for 10 min. Immunoprecipitated proteins were separated on NuPAGE 4%–12% Bis-Tris gels. After electrophoresis, gels were dried at 60 °C for 6 h and exposed to Hyperfilm in autoradiography cassettes at −80 °C for 7 or 14 days. Films were developed using standard autoradiography developer and fixer according to the manufacturer’s instructions. Anti-MDA5 reactivity was interpreted based on the presence of a radiolabelled MDA5 band at approximately 140 kDa, with reference to the positive-control plasma and molecular-weight markers.

### Pooled plasma controls and positive controls

For representative pooled readouts, pooled plasma samples were prepared by combining equal volumes of plasma from the indicated individual participants within each group. The PL-12 pool included IIM 45-47, the PL-7 pool included IIM 42-44, the SAE1 pool included IIM 52-53, the EJ pool included IIM 39-41, the Jo-1 pool included IIM 54-55, and the TIF1γ pool included IIM 48-51. Healthy control pools were prepared as follows: HC-A included HC 1-5, HC-B included HC 6-10, HC-C included HC 11-15, and HC-D included HC 16-20.

Plasma from anti-MDA5-positive patients was analysed individually. Anti-MDA5-positive patient plasma samples previously confirmed by radioisotope immunoprecipitation were used as positive controls where indicated.

### Immunocytochemistry

The HeLa cell-based immunocytochemistry assay for anti-MDA5 autoantibody detection was performed as previously described (31). HeLa cells were seeded in 24-well plates and transfected with an MDA5-expressing plasmid (pENTER-MDA5; Vigene Biosciences) using Lipofectamine 2000 transfection reagent (Invitrogen, Thermo Fisher Scientific) according to the manufacturer’s protocol. At 24 h after transfection, cells were fixed with 4% paraformaldehyde (Sigma), permeabilized with 0.3% Triton X-100, blocked. Patient plasma samples were diluted 1:5000 in PBS and incubated with transfected HeLa cells at 4 °C overnight. Bound human antibodies were detected using horseradish peroxidase-conjugated goat anti-human IgG (Jackson ImmunoResearch; cat. no. 109-035-088) diluted 1:250 in PBS, and visualized with DAB substrate (SignalStain DAB Substrate Kit, Cell Signaling Technology). Cells were counterstained with Gill II haematoxylin (Leica Biosystems), and examined by light microscopy. MDA5 expression in transfected HeLa cells was confirmed using an anti-MDA5 antibody (D74E4, Cell Signaling Technology).

### Statistical analysis

Diagnostic performance of the modified immunoprecipitation assay, line blot assay and immunocytochemistry was assessed against radioimmunoassay as the reference method. The false-positive rate was calculated as FP/(FP + TN), where FP indicates samples that were positive by the evaluated assay but negative by radioimmunoassay, and TN indicates samples that were negative by both the evaluated assay and radioimmunoassay. Cohen’s kappa values were calculated for overall and subgroup comparisons.

## Results

### Patient characteristics

A total of 93 individuals were included in this study: 55 patients with IIM, 18 suspected cases, and 20 healthy controls. Among the 55 patients with IIM, 41 had ILD and 6 had RP-ILD (**Table 1**). Using the EUROLINE Myopathies Ag kit, a line blot assay routinely used in clinical practice for anti-MDA5 antibody detection, 37 of the 55 IIM cases were classified as anti-MDA5-positive, whereas 18 were anti-MDA5-negative (**Table 1**). All 20 healthy controls tested negative for anti-MDA5 antibodies by line-blot assay, immunocytochemistry, modified immunoprecipitation, and radioimmunoassay.

**Table 1:** Anti-MDA5 test results using the line blot assay, immunocytochemistry, modified immunoprecipitation and radioimmunoassay for 55 IIM cases.

IIM No.	Line blot assay	Immuno-cytochemistry	Modified immunoprecipitation	Radio immunoassay	Age/ Gender	Clinical diagnosis
1	MDA5: 2+	positive	positive	positive	72/F	RP-ILD, CADM
2	MDA5: 3+	positive	positive	positive	50/F	CADM
3	MDA5: 3+	positive	positive	positive	62/F	ILD, CADM
4	MDA5: 3+; Ro-52: 3+	positive	positive	positive	64/F	RP-ILD, CADM
5	MDA5: 3+; Ro-52: 2+	positive	positive	positive	78/F	RP-ILD, CADM
6	MDA5: 2+	positive	positive	positive	60/F	RP-ILD, CADM
7	MDA5: 2+; Ro-52: 3+	positive	positive	positive	43/F	ILD, CADM
8	MDA5: 3+; Ro-52: 3+	positive	positive	positive	56/F	ILD, CADM
9	MDA5: 2+; Ro-52: 3+	positive	positive	positive	75/F	ILD, CADM
10	MDA5: 2+	positive	positive	positive	40/M	ILD, CADM
11	MDA5: 2+; SRP: 1+	positive	positive	positive	40/M	ILD, CADM
12	MDA5: 1+	positive	positive	positive	35/M	CADM
13	MDA5: 3+; Ro-52: 3+	positive	positive	positive	53/M	ILD, CADM
14	MDA5: 1+	negative	positive	positive	57/F	ILD, CADM
15	MDA5: 1+	positive	positive	positive	37/F	ILD, CADM
16	MDA5: 1+	positive	positive	positive	48/F	ILD, CADM
17	MDA5: 1+	positive	positive	positive	71/F	ILD, CADM
18	MDA5: 2+; Ro-52: 3+	positive	positive	positive	39/F	ILD, CADM
19	MDA5: 2+	positive	positive	positive	34/F	ILD, CADM
20	MDA5: 3+	positive	positive	positive	53/F	ILD, CADM
21	MDA5: 2+	positive	positive	positive	38/F	ILD, CADM
22	MDA5: (+) negative	positive	positive	positive	57/F	RP-ILD, CADM
23	MDA5: 3+	positive	positive	positive	63/F	ILD, CADM
24	MDA5: 2+	positive	positive	positive	51/M	ILD, CADM
25	MDA5: 1+	positive	positive	positive	43/F	ILD, CADM
26	MDA5: 1+	positive	positive	positive	58/F	ILD, CADM
27	MDA5: 3+; Ro-52: (+)	positive	positive	positive	39/M	ILD, CADM
28	MDA5: 3+	positive	positive	positive	29/F	ILD, CADM
29	MDA5: 3+; Ro-52: 3+; Jo-1: 1+	positive	positive	positive	59/F	ILD, CADM
30	MDA5: 1+	negative	positive	positive	46/F	ILD, CADM
31	MDA5: 1+; Ro-52: 2+	negative	positive	positive	48/F	ILD, CADM
32	MDA5: 3+; Ro-52: 3+; PM-Scl75: 3+	positive	positive	Positive	61/M	ILD, CADM
33	MDA5: 3+; Ro-52: 3+	positive	positive	positive	50/F	RP-ILD, CADM
34	MDA5: 2+	negative	negative	negative	53/F	PM
35	MDA5: 2+; Ro-52: 2+	negative	negative	negative	75/F	PM
36	MDA5: 2+; Ro-52: 3+; Jo-1: 3+	positive	negative	negative	42/F	ILD, Anti-synthetase syndrome(Jo-1)
37	MDA5: 1+; Jo-1: 2+; PM-Scl75: 2+	negative	negative	negative	54/F	Anti-synthetase syndrome(Jo-1)
38	MDA5: 1+; PL-7: 2+	negative	negative	negative	47/M	ILD, Anti-synthetase syndrome(PL-7)
39	EJ: 3+; Ro-52: 3+	negative	negative	negative	69/F	ILD, anti-EJ-positive DM
40	EJ: 3+; Ro-52: 3+	positive	negative	negative	47/F	ILD, anti-EJ-positive DM
41	EJ: 3+; Ro-52: 3+	positive	negative	negative	66/M	ILD, anti-EJ-positive DM
42	PL-7: 3+; Ro-52: 3+	positive	negative	negative	27/F	ILD, anti-PL-7-positive DM
43	PL-7: 3+; Ro-52: 3+	positive	negative	negative	46/F	ILD, anti-PL-7-positive DM
44	PL-7: 3+	positive	negative	negative	37/M	ILD, anti-PL-7-positive DM
45	PL-12: 3+; Ro-52: 3+	positive	negative	negative	52/F	ILD, anti-PL-12-positive DM
46	PL-12: 1+; Ro-52: 3+	negative	negative	negative	33/M	ILD, anti-PL-7-positive DM
47	PL-12: 3+	negative	negative	negative	48/F	Rheumatoid Arthritis, anti-PL-12-positive IIM
48	TIF1γ: 2+	negative	negative	negative	21/F	ILD, DM
49	TIF1γ: 1+	negative	negative	negative	49/F	ILD, DM
50	TIF1γ: 3+; Ro-52: 3+	negative	negative	negative	53/F	DM
51	TIF1γ: 2+	negative	negative	negative	28/F	DM
52	SAE1: 3+; Ro-52: 3+	negative	negative	negative	54/F	ILD, DM
53	SAE1: 1+; Ro-52: 2+	negative	negative	negative	55/F	ILD, DM
54	Jo-1: 3+; Ro-52: 3+	negative	negative	negative	61/F	ILD, anti-Jo-1-positive PM
55	Jo-1: 3+; Ro-52: 3+	negative	negative	negative	62/M	ILD, anti-Jo-1-positive PM

EJ, glycyl-tRNA synthetase; Jo-1, histidyl-tRNA synthetase; MDA5, melanoma differentiation-associated protein 5; PL-7, threonyl-tRNA synthetase; PL-12, alanyl-tRNA synthetase; Ro-52, tripartite motif-containing protein 21 (TRIM21); SAE1, small ubiquitin-like modifier activating enzyme 1; TIF1γ, transcriptional intermediary factor 1 gamma. IIM, idiopathic inflammatory myopathy; ILD, interstitial lung disease; RP-ILD, rapidly progressive interstitial lung disease; DM, dermatomyositis; CADM, clinically amyopathic dermatomyositis; PM, polymyositis. Anti-ARS, anti-aminoacyl-tRNA synthetase antibody. The clinical diagnosis column reflects the final diagnosis assigned by the treating rheumatologists after complete clinical evaluation. Anti-ARS antibody positivity alone was not used to classify patients as having antisynthetase syndrome unless compatible clinical criteria were fulfilled.

In this study, the 18 suspected cases were defined as non-IIM patients who tested positive for anti-MDA5 by commercial line-blot assay but did not have a final diagnosis of IIM, DM, PM, or CADM after rheumatologic evaluation. These suspected cases tested positive for anti-MDA5 by line blot using the EUROLINE Myopathies Ag kit, but were subsequently diagnosed with other autoimmune or non-IIM conditions (**Table 2**). Some of these cases had clinical features overlapping with anti-MDA5-associated disease, including ILD or vasculitic manifestations. The clinical features, final diagnoses, and autoantibody profiles of the IIM and non-IIM groups are summarised in **Tables 1**, **2**. To compare diagnostic performance across assays, anti-MDA5 antibodies were evaluated using radioimmunoassay, immunocytochemistry, and modified immunoprecipitation, with radioimmunoassay used as the reference standard.

**Table 2:** Anti-MDA5 testing results in non-IIM patients with line-blot anti-MDA5 positivity.

Susp No.	Line blot assay	Immuno-cytochemistry	Modified immuno-precipitation	Radio immunoassay	Age/ Gender	Clinical diagnosis
1	MDA5: 1+; Ro-52: 3+	negative	negative	negative	67/F	ILD, Sjogren’s Syndrome
2	MDA5: 1+; PM-Scl75: 1+	negative	negative	negative	65/F	Mixed Connective Tissue Disease
3	MDA5: 1+; Ro-52: 3+; PL-12: 1+	positive	negative	negative	73/F	ILD, Systemic Lupus Erythematosus
4	MDA5: 1+; Ro-52: 3+	positive	negative	negative	54/F	Rheumatoid Arthritis
5	MDA5: 1+	negative	negative	negative	49/M	Systemic Lupus Erythematosus
6	MDA5: 1+	negative	negative	negative	53/F	Seronegative rheumatoid arthritis
7	MDA5: 1+	positive	negative	negative	47/F	Osteoarthritis
8	MDA5: 3+; Ro-52: 3+; Mi-2α: 1+	negative	negative	negative	38/F	Systemic Lupus Erythematosus
9	MDA5: 1+	negative	negative	negative	44/F	Mixed Connective Tissue Disease
10	MDA5: 1+	negative	negative	negative	51/F	Systemic Sclerosis
11	MDA5: 2+; Ro-52: 3+; TIF1γ: 1+	negative	negative	negative	69/F	Sjogren’s Syndrome
12	MDA5: 1+; Ro-52: 3+	negative	negative	negative	29/F	Mixed Connective Tissue Disease
13	MDA5: 2+; TIF1γ: 2+	negative	negative	negative	49/M	Seronegative rheumatoid arthritis
14	MDA5: 1+	negative	negative	negative	43/F	Seronegative rheumatoid arthritis
15	MDA5: 2+	positive	negative	negative	46/F	Sjogren’s Syndrome
16	MDA5: 2+; PL-7: 1+	negative	negative	negative	52/F	Breast Cancer
17	MDA5: 3+; Ro-52: 3+	positive	negative	negative	21/F	ILD, Vasculitis
18	MDA5: 1+; Ro-52: 1+; NXP2: (+); SAE1: 1+	positive	negative	negative	61/M	Chronic Obstruction Pulmonary Disease

MDA5, melanoma differentiation-associated protein 5; Mi-2α, chromodomain-helicase-DNA-binding protein 3 (CHD3); NXP2, nuclear matrix protein 2; PM-Scl75, polymyositis-scleroderma 75-kDa protein; PL-7, threonyl-tRNA synthetase; PL-12, alanyl-tRNA synthetase; Ro-52, tripartite motif-containing protein 21 (TRIM21); SAE1, small ubiquitin-like modifier activating enzyme 1; TIF1γ, transcriptional intermediary factor 1 gamma; ILD, interstitial lung disease.

### Radioimmunoassay-based classification reveals false-positive anti-MDA5 results by routine screening assays

**Figure 1:**
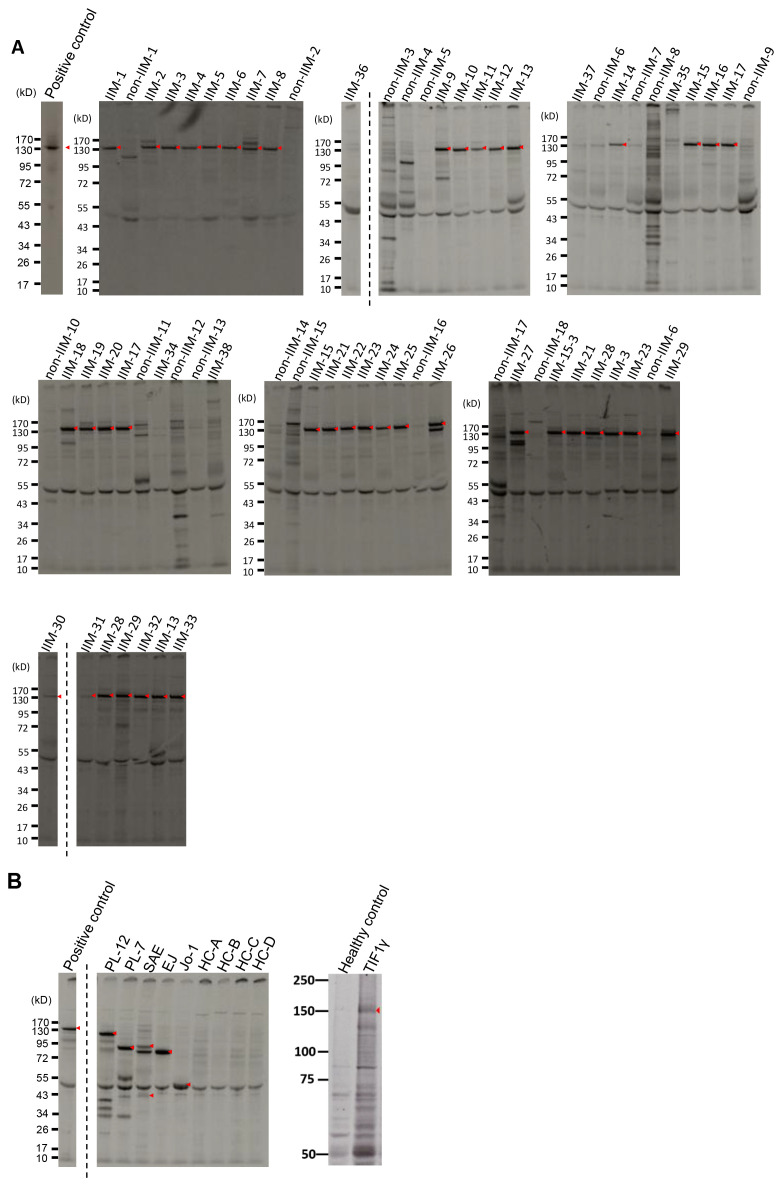
Radioimmunoassay for anti-MDA5 antibody detection using [^35^S]methionine-labelled THP-1 cell lysates. **(A)** Immunoprecipitation of radiolabelled MDA5 using plasma from individual IIM cases and non-IIM line-blot anti-MDA5-positive cases. MDA5 was detected as a dominant band at approximately 140 kDa, with an occasional higher band at approximately 155 kDa. Molecular-weight markers were included in each gel. Anti-MDA5-positive patient plasma samples previously confirmed by radioisotope immunoprecipitation were used as positive controls where indicated. **(B)** Representative control lanes using pooled plasma from IIM cases with non-MDA5 autoantibodies and HCs, showing the absence of specific anti-MDA5 reactivity. Equal volumes of plasma from the indicated individuals were pooled as follows: PL-12 (IIM 45-47), PL-7 (IIM 42-44), SAE1 (IIM 52-53), EJ (IIM 39-41), Jo-1 (IIM 54-55), TIF1γ (IIM 48-51), HC-A (HC 1-5), HC-B (HC 6-10), HC-C (HC 11-15), and HC-D (HC 16-20). Positive-control plasma was included as indicated.

We first used radioimmunoassay, the laboratory reference method in this study, to classify anti-MDA5 status across the cohort. To improve detection of isotope-labelled MDA5 in cell lysates, we modified the conventional radioimmunoassay protocol by replacing K562 cells with THP-1 cells and inducing MDA5 expression with LPS during 35S labelling. This approach enabled detection of anti-MDA5 antibodies in patient plasma by immunoprecipitation of 35S-labelled MDA5 using fewer cells and without exogenous MDA5 overexpression (**Figure 1**). MDA5 was detected as a dominant band with a molecular weight of 140 kDa (**Figure 1A**), and a less frequent form of 155 kDa was also observed (**Figure 1A**, IIM patient 26), consistent with previous reports (7). Among the 55 IIM cases, 37 were positive by line blot. Of these, 32 were confirmed to be anti-MDA5-positive by radioimmunoassay, whereas 5 were not confirmed, indicating false-positive line blot results. These false-positive cases included 2 PM cases and 3 anti-synthetase syndrome cases (IIM-34 to IIM-38). In addition, 1 line blot-negative IIM case (IIM patient 22) was positive by radioimmunoassay, giving a total of 33 radioimmunoassay-confirmed anti-MDA5-positive IIM cases. By contrast, none of the non-IIM cases showed a clearly positive anti-MDA5 signal by radioimmunoassay (**Figures 1A, B**). IIM cases with other myositis-related autoantibodies and HCs were also negative, supporting the specificity of the anti-MDA5 signal detected by this assay (**Figure 1B**).

### A modified non-radioactive immunoprecipitation assay provides concordant and cleaner confirmatory readouts

**Figure 2:**
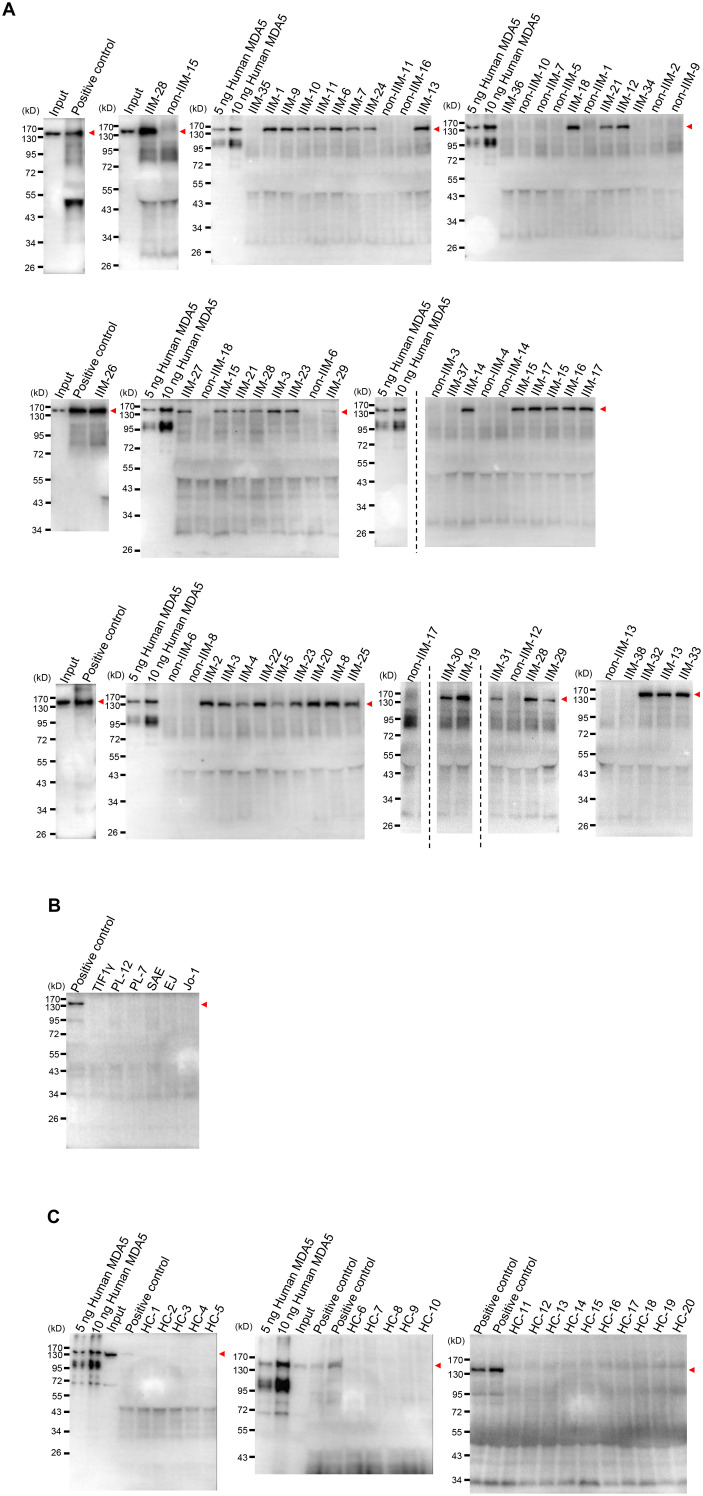
Modified immunoprecipitation followed by immunoblotting for anti-MDA5 antibody detection using LPS-stimulated THP-1 cell lysates. Plasma samples were used for immunoprecipitation from LPS-stimulated THP-1 cell lysates, and immunoprecipitated MDA5 was detected by immunoblotting with an anti-MDA5 antibody. **(A)** Detection of immunoprecipitated MDA5 using plasma from individual IIM cases and non-IIM line-blot anti-MDA5-positive cases. Recombinant human MDA5 protein (5 ng and 10 ng), input lysate, and anti-MDA5-positive patient plasma controls were included as indicated. **(B)** Representative specificity-control lanes using pooled plasma from IIM cases with non-MDA5 autoantibodies, including PL-12, PL-7, SAE1, EJ, Jo-1 and TIF1γ. **(C)** Representative healthy control plasma samples (HC-1 to HC-20). For pooled control lanes, equal volumes of plasma from the indicated individuals were combined as described in **Figure 1B**. Positive-control plasma and input lysate were included as indicated.

We next evaluated the modified immunoprecipitation assay as a non-radioactive confirmatory method for anti-MDA5 antibody detection by immunoblot analysis of immunoprecipitated MDA5 using LPS-stimulated THP-1 cell lysates (**Figure 2**). In contrast to radioimmunoassay, this assay generated a clear and readily interpretable signal with minimal background reactivity. In the IIM group, the modified immunoprecipitation assay showed complete concordance with radioimmunoassay for detection of anti-MDA5 antibodies, identifying the same positive cases while providing a cleaner readout. These findings indicate that the modified assay reproduced the reference-method classification without the need for radioactive detection.

The confirmatory value of the modified immunoprecipitation assay was particularly evident in the non-IIM line-blot anti-MDA5-positive cases. Although none of these cases showed a clearly positive anti-MDA5 signal by radioimmunoassay, interpretation was complicated in some samples by substantial background reactivity, including non-IIM cases 11 and 16 (**Figure 1**). In contrast, the modified immunoprecipitation assay showed no corresponding background signal in these cases (**Figure 2A**), allowing clearer discrimination between true-negative and potentially misleading results. In addition, IIM cases with other myositis-related autoantibodies and healthy controls were negative in the modified immunoprecipitation assay (**Figures 2B, C**), further supporting assay specificity.

Across the entire cohort, the modified immunoprecipitation assay identified 33 true positives and 60 true negatives, with no false-positive or false-negative results. This corresponded to a false-positive rate of 0% and complete concordance with radioimmunoassay (kappa = 1.0) in this cohort (**Table 3**). Taken together, these results show that the modified immunoprecipitation assay not only matched radioimmunoassay in anti-MDA5-positive cases, but also provided a clearer and more specific readout in diagnostically challenging samples. These features support its role as a practical confirmatory assay for anti-MDA5 antibody detection.

**Table 3:** Comparison of each test result (line blot assay, immunocytochemistry and modified immunoprecipitation) with the reference standard (radioimmunoassay) in all cases.

(a) Line blot assay versus radioimmunoassay							
		Radioimmunoassay			Line-blot assay efficiency		
		Positive	Negative	Total			
Line blot assay	Positive	32	23	55	False positive rate		Cohen’s κ
	Negative	1	37	38			
	Total	33	60	93	38.33%		0.51
(b) Immunocytochemistry versus radioimmunoassay							
		Radioimmunoassay			Immunocytochemistry efficiency		
		Positive	Negative	Total			
Immuno-cytochemistry	Positive	30	13	43	False positive rate		Cohen’s κ
	Negative	3	47	50			
	Total	33	60	93	21.67%		0.65
(c) Modified immunoprecipitation versus radioimmunoassay							
		Radioimmunoassay			Modified immunoprecipitation efficiency		
		Positive	Negative	Total			
Modified Immuno-precipitation	Positive	33	0	33	False positive rate		Cohen’s κ
	Negative	0	60	60			
	Total	33	60	93	0%		1

### Routine assays remained less specific than the modified immunoprecipitation assay

**Figure 3:**
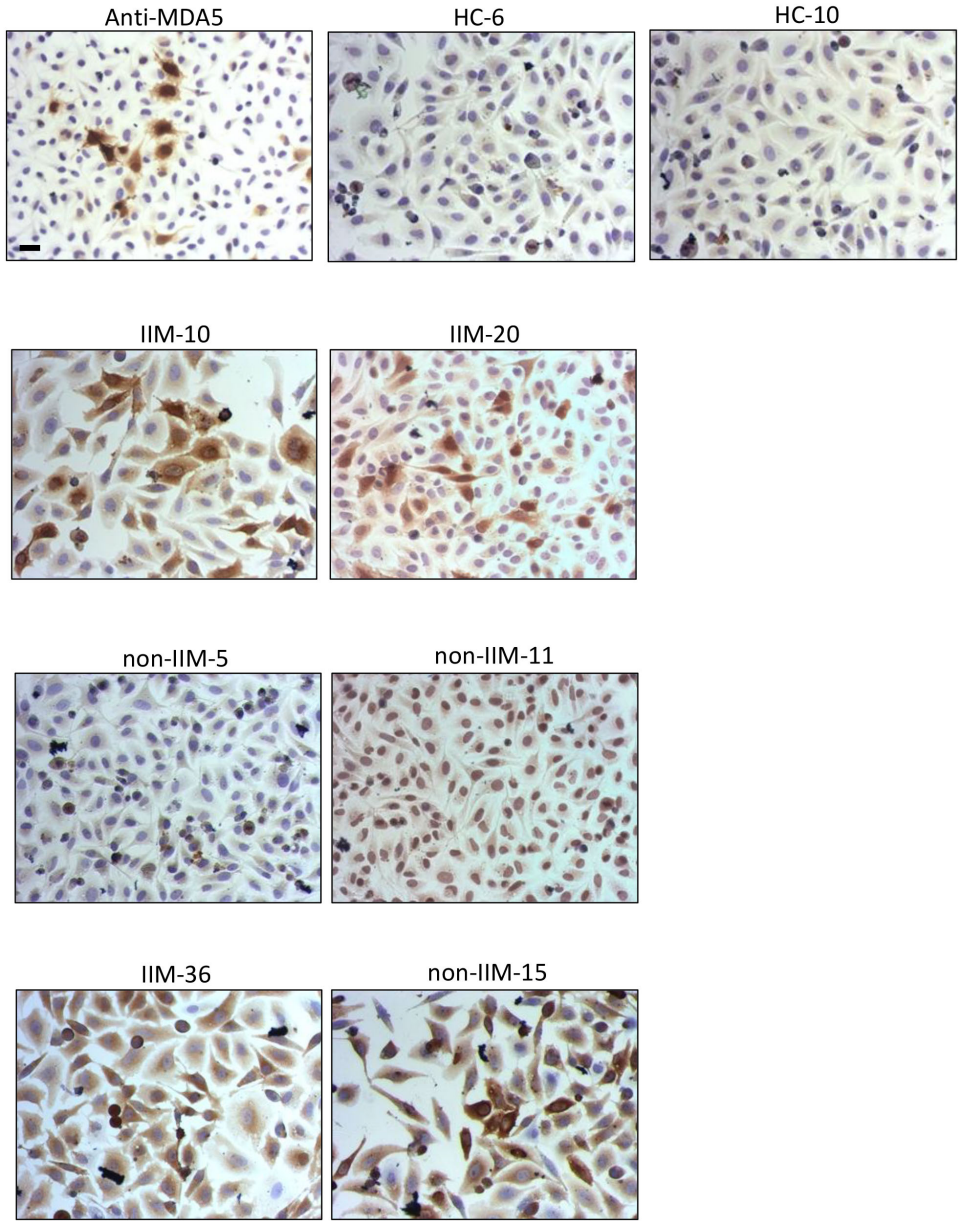
Immunocytochemical detection of anti-MDA5 reactivity in MDA5-transfected HeLa cells. **(A)** MDA5 expression in transfected HeLa cells was confirmed using an anti-MDA5 antibody as a positive control. Representative healthy controls (HC-6 and HC-10) are shown for comparison. **(B)** Representative true-positive IIM cases (IIM-10 and IIM-20) showing positive immunocytochemical staining. **(C)** Representative true-negative non-IIM line-blot anti-MDA5-positive cases (non-IIM cases 5 and 11) showing no specific immunocytochemical staining. **(D)** Representative false-positive immunocytochemical staining in an IIM case (IIM-36) and a non-IIM line-blot anti-MDA5-positive case (non-IIM case 15), both of which were negative by modified immunoprecipitation and radioimmunoassay. Brown DAB staining indicates anti-MDA5 reactivity, and nuclei were counterstained with haematoxylin. Scale bar = 20 μm.

We next compared the performance of line blot and immunocytochemistry against radioimmunoassay across the full cohort, including both IIM patients and non-IIM line-blot anti-MDA5-positive cases. Immunocytochemistry detected anti-MDA5 reactivity in a proportion of positive cases, but its interpretation was limited by false-positive staining in both groups (**Figure 3**). This was particularly evident in cases such as IIM-36 and non-IIM case 15, which were positive by immunocytochemistry despite being negative by radioimmunoassay. These findings indicate that immunocytochemistry, although capable of identifying some anti-MDA5-positive cases, lacks sufficient specificity for confident discrimination between true-positive and false-positive results.

We therefore quantified the diagnostic performance of line blot and immunocytochemistry in all cases (**Table 3**). Across the entire cohort, immunocytochemistry identified 30 true positives, 47 true negatives, 13 false positives, and 3 false negatives, corresponding to a false-positive rate of 21.67% and moderate agreement with radioimmunoassay (κ = 0.65). By comparison, line blot identified 32 true positives, 37 true negatives, 23 false positives, and 1 false negative, yielding a substantially higher false-positive rate of 38.33% and lower agreement with radioimmunoassay (κ = 0.51). Thus, when all cases were considered together, both methods showed limited specificity, with line blot generating the highest number of false-positive anti-MDA5 results.

Because the line blot kit is routinely used in clinical practice primarily in the evaluation of IIM, we further examined assay performance within the IIM subgroup alone (**Table 4**). In this setting, line blot performed better than immunocytochemistry, identifying 32 true positives, 17 true negatives, 5 false positives, and 1 false negative, corresponding to a false-positive rate of 22.73% and substantial agreement with radioimmunoassay (κ = 0.77). Immunocytochemistry identified 30 true positives, 15 true negatives, 7 false positives, and 3 false negatives, with a false-positive rate of 31.82% and moderate agreement (κ = 0.61). These findings suggest that line blot has greater diagnostic utility than immunocytochemistry within its intended clinical setting, but still misclassified a meaningful proportion of IIM cases.

**Table 4:** Comparison of each test result (line blot assay and immunocytochemistry) with the reference standard (radioimmunoassay) in IIM cases.

(a) Line blot assay versus radioimmunoassay						
		Radioimmunoassay			Line blot assay efficiency	
		Positive	Negative	Total		
Line blot assay	Positive	32	5	37	False positive rate	Cohen’s κ
	Negative	1	17	18		
	Total	33	22	55	22.73%	0.77
(b) Immunocytochemistry versus radioimmunoassay						
		Radioimmunoassay			Immunocytochemistry efficiency	
		Positive	Negative	Total		
Immuno-cytochemistry	Positive	30	7	37	False positive rate	Cohen’s κ
	Negative	3	15	18		
	Total	33	22	55	31.82%	0.61

Taken together, these results show that false-positive anti-MDA5 results are common with routine assays, particularly when testing is extended to clinically ambiguous cases or to patients who do not have definite IIM. Even within the IIM subgroup, neither line blot nor immunocytochemistry achieved the accuracy observed with the modified immunoprecipitation assay. These data underscore the need for a more specific confirmatory method for anti-MDA5 antibody detection in clinical practice.

## Discussion

In this study, we performed a comparative evaluation of currently used methods for anti-MDA5 antibody detection and developed a modified, non-radioactive immunoprecipitation assay as a practical confirmatory method. Three findings are particularly important. First, false-positive anti-MDA5 results were common with routine screening assays, particularly line blot, and were especially frequent in clinically suspected cases and in patients who were ultimately diagnosed with other autoimmune or non-IIM conditions. Second, although line blot performed better than immunocytochemistry within the IIM subgroup, neither assay matched the specificity of the modified immunoprecipitation assay. Third, the modified immunoprecipitation assay showed complete concordance with radioimmunoassay in this cohort while providing a cleaner and more readily interpretable readout. These findings are clinically relevant because anti-MDA5 antibodies are strongly associated with ILD, particularly RP-ILD, in DM and CADM, and laboratory misclassification may therefore influence disease interpretation and downstream clinical decision-making (7, 17–21).

The translational value of this work lies not only in the comparative evaluation of assay performance, but also in the establishment of a THP-1/LPS-based modified immunoprecipitation assay as a practical confirmatory method for anti-MDA5 antibody detection in clinically ambiguous cases. MDA5 is a cytosolic viral RNA sensor that is predominantly expressed in monocytes and macrophages and is regulated during innate immune activation (16, 32, 33). Building on this biology, we replaced K562 cells with THP-1 cells and induced MDA5 expression with LPS, thereby increasing the availability of endogenous antigen for immunoprecipitation. In contrast to earlier approaches that relied on isotope-labelled material and large cell numbers (7, 17, 34), our modified assay uses LPS-stimulated THP-1 lysates combined with immunoblot detection, allowing clear visualization of immunoprecipitated MDA5 without radioactive detection in the confirmatory step. This design strengthens the potential feasibility of the assay as a second-line laboratory approach when routine anti-MDA5 results are difficult to interpret.

Our findings also reinforce growing concerns regarding interpretation of commercial myositis antibody assays. Previous studies have shown that the reliability of immunoassays in myositis depends on antibody specificity and clinical context, and that line blot positivity may have limited positive predictive value, particularly when testing is performed outside a well-defined IIM population (22–24). In our cohort, this issue was particularly evident in the clinically suspected cases, all of whom were initially positive by line blot but were not confirmed by radioimmunoassay. Even within the IIM subgroup, line blot still produced false-positive results, although its performance was better than that of immunocytochemistry. Accordingly, our data do not argue against the use of line blot as an initial screening tool in the appropriate clinical setting; rather, they support the use of confirmatory testing when positive anti-MDA5 results are discordant with the overall clinical phenotype or when testing is extended to patients with broader autoimmune or pulmonary presentations.

Some non-IIM line-blot anti-MDA5-positive cases were also positive by immunocytochemistry but negative by both radioimmunoassay and modified immunoprecipitation. These cases were therefore interpreted as unconfirmed anti-MDA5 results, or false positives relative to the reference radioimmunoassay. The concordant false-positive findings by line blot and immunocytochemistry may reflect shared susceptibility of these immunochemical screening assays to nonspecific or cross-reactive plasma IgG binding, particularly in autoimmune sera with multiple autoantibodies. In contrast, radioimmunoassay and modified immunoprecipitation provide biochemical confirmation of MDA5 reactivity by detecting immunoprecipitated MDA5 protein, allowing clearer discrimination between true anti-MDA5 reactivity and potentially misleading background signals.

An important strength of the modified immunoprecipitation assay was its performance in diagnostically challenging samples. In non-IIM line-blot anti-MDA5-positive cases with substantial background reactivity by radioimmunoassay, the modified assay yielded a clearer negative readout without the nonspecific background that complicated interpretation. This suggests that the assay adds value not only by reproducing radioimmunoassay-based classification, but also by improving confidence in result interpretation when conventional methods are ambiguous. In this respect, the assay may be particularly useful as a second-line confirmatory test for patients with positive line-blot results but uncertain clinical context. Such an approach could be especially relevant in the evaluation of ILD, where anti-MDA5 positivity may substantially influence perceived risk of RP-ILD and the urgency of treatment escalation (7, 18, 21, 34).

This study has limitations. The cohort size was modest, the number of confirmed anti-MDA5-positive cases was limited, and all samples were obtained from a single centre. In addition, the assay was evaluated in a retrospective comparative framework rather than in a prospective clinical testing pathway. Although complete concordance between the modified immunoprecipitation assay and radioimmunoassay was observed in this cohort, validation in larger, independent and ideally multicentre cohorts is required to confirm the generalizability and clinical applicability of this non-radioactive assay. Further studies should also assess inter-laboratory reproducibility, technical standardization and the practical role of the assay within a stepwise anti-MDA5 testing process.

In conclusion, our study highlights the need for highly specific confirmatory testing in anti-MDA5 antibody detection. False-positive results were common with line blot and immunocytochemistry, particularly outside definite IIM, whereas the modified immunoprecipitation assay showed complete concordance with radioimmunoassay and provided a clear, non-radioactive readout. Taken together, these findings support the modified immunoprecipitation assay as a clinically relevant confirmatory method for anti-MDA5 antibody detection that warrants further validation in larger and independent cohorts.

## Data Availability

The original contributions presented in the study are included in the article/supplementary material. Further inquiries can be directed to the corresponding authors.
